# Vision and Quality of Life: Development of Methods for the VisQoL Vision-Related Utility Instrument

**DOI:** 10.1080/09286580801979417

**Published:** 2008-09-09

**Authors:** Stuart Peacock, RoseAnne Misajon, Angelo Iezzi, Jeff Richardson, Graeme Hawthorne, Jill Keeffe

**Affiliations:** ^1^Centre for Health Economics in Cancer, British Columbia Cancer Agency, Vancouver, British Columbia, Canada; ^2^Department of Health Care and Epidemiology, University of British Columbia, Vancouver, Canada; ^3^School of Political and Social Inquiry, Monash University, Melbourne, Australia; ^4^Centre for Health Economics, Monash University, Melbourne, Australia; ^5^Department of Psychiatry, University of Melbourne, Melbourne, Australia; ^6^Centre for Eye Research Australia, Department of Ophthalmology, University of Melbourne, Melbourne, Australia; ^7^Vision CRC, University of New South Wales, Sydney, Australia

**Keywords:** Vision-related quality of life, utility instrument, cost-utility analysis, economic evaluation, time trade-off

## Abstract

**Purpose:**

To describe the methods and innovations used in constructing the VisQoL, a vision-related utility instrument for the health economic evaluation of eye care and rehabilitation programs.

**Methods:**

The VisQoL disaggregates vision into six items. Utilities were estimated for item worst responses (the worst level for each item, with all other items at their best level) and VisQoL all-worst responses (all items at their worst level) using the time trade-off procedure. Time trade-off questions require people to imagine living a fixed number of years with a particular health condition and then indicate how many of those years of life they would be willing to trade to have perfect health. Where respondents indicated a health state was “worse than death” negative utilities were estimated. Time trade-off questions minimized the “focusing effect,” which occurs if respondents discount the fact that all other aspects of health are at their best when answering questions, by using pictorial and verbal aids.

**Results:**

Item utilities were combined using a multiplicative model, and VisQoL model utilities placed on a scale where 0.00 and 1.00 represent full health and death, respectively. The VisQoL allows utilities to be calculated for a wide range of vision-related conditions.

**Conclusion:**

The 6-item VisQoL has excellent psychometric properties and is specifically designed to be sensitive to vision-related quality of life. It is the first instrument to permit the rapid estimation of utility values for use in economic evaluations of vision-related programs.

## Introduction

The overarching aim of eye-care and vision-related rehabilitation programs is to improve the quality of life (QoL) of visually impaired people, but resources for these programs are limited.^1^ Vision impairment has a significant impact on length and QoL.^2^,^3^ Previous research has shown that vision impairment is associated with an increased risk of falls, hip fractures, depression, social isolation, greater need for community services and greater risk of admission to nursing homes.^2^,^4^–^8^ In a limited resource environment, it is critical to assess the value of health care programs in terms of both their costs and outcomes—improvements in length and quality of life—using economic evaluation methods. The purpose of this article is to describe the methods and innovations used in constructing the VisQoL—a vision-related utility instrument for the health economic evaluation of eye care and rehabilitation programs.

In an earlier paper, we outlined the need for a vision-related utility instrument to measure gains in health-related quality of life (HRQoL).^9^ Utility instruments quantify the strength of people’s preferences (utility) for a health state. This information is used to construct Quality Adjusted Life Years (QALYs) by multiplying the relevant number of life years by the index of utility. The QALY may then be used to measure the effect of a health intervention upon the quality and length of life. In cost utility analysis (CUA) this benefit is compared with the cost of the intervention. Cost utility analysis therefore has an important strength: health gains from programs for different diseases or conditions can be directly compared, allowing rational decisions about the allocation of scarce health care programs to be made. However, whilst generic utility instruments—such as the AQoL,^10^,^11^ EQ-5D,^12^ or HUI-III^13^—may be used to estimate QALY gains, they may not be sufficiently sensitive to vision-related QoL^14^,^15^ limiting their usefulness in the evaluation of vision-related programs.

To overcome this problem, a “vision enhanced” generic instrument is needed; that is, a generic instrument which has been adapted to have greater discriminatory power (sensitivity) in the health states most relevant for the measurement of visual impairment. No such instrument is currently available.^9^ Given that the impact of vision on QoL is multi-faceted, a multi-attribute utility (MAU) instrument is required to accurately describe the full effect of vision upon HRQoL. Such an MAU instrument should be constructed using standard psychometric procedures to ensure reliability, construct validity, be sensitive to as much of the full range of HRQoL as is practical, and have good predictive validity.^16^,^17^

The aim of this project was to develop a multi-attribute vision-related utility measure referred to as the Vision and Quality of Life Index (VisQoL). The purpose of this article is to describe the methodology used to develop utilities for the VisQoL instrument.

## The VisQoL Descriptive System

The construction of the 6 item VisQoL descriptive system is presented in detail elsewhere.^9^ The VisQoL is based upon the hypothesis that (dis)utility depends primarily upon the effects of a health condition upon a person’s capacity to achieve a productive and fulfilling life in their social context. To confirm the final VisQoL model, the 6 items were administered to a total of 374 participants; 39% with a vision impairment. A pooled structural equation modeling (SEM) analysis with good statistical power showed the model to have very good fit properties (root mean square error of approximation = 0.0010).^9^ Items from the short 6-item VisQoL are presented in the appendix.

## Utility Scaling

In order to derive utilities for all health states described by the VisQoL, MAU theory requires that health states be decomposed into their constituent items and the utilities estimated for each item in isolation. To obtain the overall utility for a multi-attribute health state—a health state described by the six VisQoL items— the utilities for the items must then be recombined into a single index, using an appropriate model. The model may either be derived by econometric analysis of the observed utilities or by the use of decision analytic techniques to fit a simple additive or a multiplicative model. The fully scaled MAU instrument may then be used to estimate the utility of VisQoL health states.

### Issues in Weighting the VisQoL

There are three main techniques for measuring utilities: the time trade-off (TTO); the standard gamble (SG); and the Rating Scale (RS).^18^,^19^ Time trade off requires people to imagine living a fixed number of years with a particular health condition, and then indicate how many of those years of life they would be willing to trade to have perfect health. SG determines the risk of death people are willing to take for a treatment that would give them perfect health. RS requires people to imagine living with a particular health condition, and then indicate how good or bad that health state is on a 0–100 scale (where perfect health is marked 100 and dead, or the worst health state they can imagine, is marked 0). However, because RS does not explicitly involve trade-offs, some authors have questioned whether the RS method produces utilities which are consistent with economic theory.

The VisQoL adopted the TTO scaling procedure. This has increasingly been the procedure of choice in cost utility studies. It produces scores of the same magnitude as the SG, and as Richardson^20^ argued, the TTO produces measures of utility that are theoretically more satisfactory for inclusion in cost utility analysis. Utility scores are estimated from TTO questions using equation (1). Respondents are asked to select the number of years, *x*, living in full health that would be equivalent to living *n* years in a health state with a utility score, U, which is less than full health. This allows the calculation of utility as *x/n*. In the VisQoL a time period *n* = 10 was adopted, consistent with methods developed for the AQoL.^10^,^11^ This time period was chosen for three reasons. Some authors have suggested a time period of “the rest of your lifetime,” but this may bias results as TTO values may be related to how long respondents think they will live. Some respondents find long time periods, such as 20 or 30 years, to be unrealistic given how long they think they will live. Conversely, short time periods, such as 1 or 2 years, may result in many respondents failing to make trade-offs between quality and length of life.


[1] U=x/n

**Figure 1:**
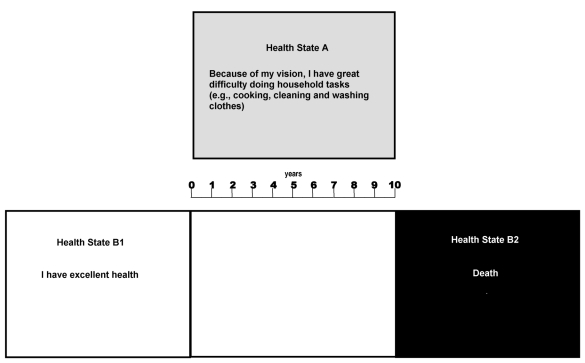
An example of a visual aid for time trade off questions.

An example of a visual aid for TTO questions is shown in Figure 1. The bottom half of the figure represents a slide with the black section representing death. This was moved left and right in order to “flip-flop” the number of years of full health until they were equivalent to 10 years in the health state depicted. While the respondent was asked to visualize the poor health state they were visually and verbally reminded that other aspects of their health were at their best level.

There are health states where people would prefer to be dead, that is, there are negative utilities. The transformation of negative scores is discussed in Richardson and Hawthorne.^21^ It was argued that the common approach of adopting -1.00 as the lower boundary in order to achieve “symmetry” with positive utilities is unconvincing. While the numerical score is “symmetrical, the logic behind the score is different. More importantly, it is inconceivable that people can experience such a health state. Richardson and Hawthorne^21^ conclude that negative utilities should be given a limiting score of −0.25 which is equivalent to a disutility (DU) score, DU = 1.25. This convention was also adopted for the VisQoL. A simple mathematical function is then used to transform all negative scores so that “values” between zero and minus infinity are constrained to the range 0.00 to −0.25, which is equivalent to constraining negative utility values to the range 1.00 to 1.25.

The TTO protocol for scaling the AQoL and VisQoL included a further important innovation. Scaling questions were designed to minimize error arising from the “focusing effect.”^22^,^23^ The focusing effect is a particular threat to the validity of decomposed, then reconstructed, instrument scores. It occurs if respondents forget, or discount, the fact that all other aspects of health are at their best when answering scaling questions, and there is a disproportionate focus upon the single part of a health state which is poor. For example, a respondent who is asked to rate life in a wheelchair may easily forget that with good communication, no pain and good health in all other respects, and a social environment which allows them to be relatively independent, it is possible to enjoy a relatively good life. To overcome this problem, an overview of the full health state was included in the protocol which indicated which of the items were at their item worst or item best levels, or at a given intermediate level between worst and best. This took the form of a visual aid and verbal reminders. When a respondent was asked to focus upon poor health in one item only, they were provided this information pictorially and verbally to reminded them that other items were good or at their all best.

## Modeling Utilities

The VisQoL has a simple structure, with vision disaggregated into six items. The recombination of items requires estimation of the item weights. MAU theory suggests that when the sum of importance weights exceeds unity a multiplicative model should be used.^24^ This model is also important in the context of health state utilities because it is possible for a number of health states to independently impact catastrophically upon the quality of life. For example, intense pain and intense depression may both reduce the quality of life to zero. This cannot be described in a simple additive model where importance weights must sum to unity and, consequently, the importance weights on depression and upon pain must be numerically small. In contrast, the multiplicative model permits any dimension to reduce QoL to a level equivalent to death. The VisQoL multiplicative MAU model is:

[2] $$U=U_{1}^{*}U_{2}^{*}U_{3}^{*}U_{4}^{*}U_{5}^{*}U_{6}$$

where *U* is the utility of the combined multi-attribute health state and *U_i_* are the utility scores for items. The actual model is somewhat more flexible. It is calculated using disutilities rather than utilities and these are adjusted for the relative importance of each of the model’s items. This results in equation (3) in which *x_i_* are the item scores, *w_i_* are the item importance weights, and *k* is a scaling constant. This is obtained by solving equation (4) for *k*, which is similar to the requirement in an additive model that the dimension weights sum to unity. (Note that solving this equation for *k* requires no additional empirical data—the scaling constant is determined only by the importance weights). The relationship between utility and disutility is given in equation (5) where DU is the disutility score corresponding with utility U.

[3] $$DU=\frac{1}{k}\left[\prod_{i=1}^{n}[1+kw_{i}DU_{i}(x_{i})]-1\right]$$

[4] $$k=\prod_{i=1}^{n}\left(1+kw_{i}\right)-1$$

[5] U=1−DU

**Figure 2:**
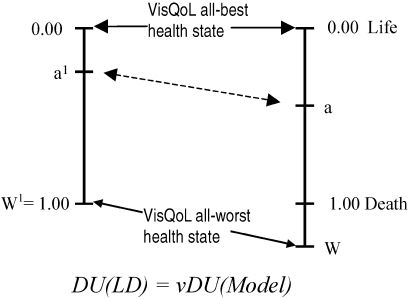
Mapping “model” onto “life-death” utilities. VisQol = DU(LD)-rescaled disutility measured on a best health to death scale; Vision and Quality of Life Index; DU = disutility.

Insertion of the item weights, *w_i_*, into equation (3) produces disutility scores constrained to the range 0.00–1.00. For the VisQoL model these endpoints correspond with the VisQoL all-best and all-worst health state respectively. As the worst health state is not necessarily death, it is necessary to map “model disutility” onto a second scale in which 0.00 and 1.00 represent full health and death respectively. Recalibrating the instrument to a VisQoL all-best to death scale (where death DU = 1.00 or, more simply utility has a value of 0.00), requires the multiplication of the model scores by *v*, the disutility of the VisQoL all-worst health state measured on a life-death scale (equation 6):

[6] DU(LD)=ν.DU(Model)

where DU is disutility measured on a VisQoL all-best and all-worst scale calculated from equation (3), and DU(LD) is the rescaled disutility measured on a best health to death (often referred to as a life-death) scale. The transformation is illustrated in Figure 2.

This task is straightforward when the model involves disutilities (and it is partly for this reason that modeling is conducted in terms of disutility scores). Establishing a single “bridge” or equivalence between any two corresponding points and *a*^1^ and *a* on the “model” and “life-death” scales respectively will permit the recalibration of the model utility values using the equation:

[7] $$U(LD)=(\frac{a}{a^{1}})U(\text{Model})$$

The VisQoL all-worst health state, *W*, is an obvious choice for calculating the bridge. Finally, for equation 8 to yield correct values for disutilities on the life-death scale, it is necessary to replace the item weights *w_i_* with adjusted weights *w_i_*/*W* in equation (3). Utilities measured on the life-death scale are calculated by inserting the recalibrated DU(LD) values from equation (6) into equation (5).

## Estimating Utilities from Survey Data

Face-to-face TTO interviews were conducted by an interviewer to obtain VisQoL utilities. TTO results were obtained for key parameters: item worst responses (the worst level or response category for each item, with all other items at their best level); and, VisQoL all-worst responses (all items at their worst level). Ethics approval was granted by the Royal Victorian Eye and Ear Hospital Human Research and Ethics Committee and the tenets of the Declaration of Helsinki were adhered to.

Scaling the VisQoL also required utilities for each of the remaining 28 intermediate item responses (item levels when items are not at their worst level). However, if a single respondent was asked to provide all of the information required using TTO questions, the interview burden would have been excessive (it would require 35 separate TTO questions). Consequently, interviews were used to collect relatively complex TTO scores for the major parameters, *viz*, item worst responses and VisQoL all-worst responses. Scores for intermediate item responses were collected using 6 RS questions. RS questions have the advantage that they are simpler to administer than TTO questions, and place less cognitive burden on the respondent as respondents are already familiar with the principles of scaling. There is precedent for such an approach. The HUI-III used a combination of TTO and RS techniques to estimate utilities for the instrument.^25^,^26^ Similarly, the first SF-6D utility instrument used a combination of SG and RS techniques to estimate utilities.^27^

Item levels were constructed to achieve two objectives. The first was to obtain item level responses that are approximately equidistant between the item best and worst health state. Thus, for example, if the utility scores for item levels assumed values of 0.00, 0.02, 0.04, 0.06, and 1.00, then the item would be unable to detect changes in the range 0.06–1.00. The second objective was to obtain greater sensitivity near full health than has been achieved in previous utility instruments. Item level scores were measured on a disutility scale where the endpoints are the item best (DU = 0.00) and the item worst (DU = 1.00). Where RS scores are used in instrument construction, a transformation is required based on TTO or SG.^25^–^27^ RS scores for item responses were transformed into TTO equivalent scores using a two-part transformation function described in Richardson et al.^23^

Item worst scores, *w_i_*, were estimated from TTO results and were measured on a scale from VisQoL all-best (DU = 0.00) to VisQoL all-worst (DU = 1.00). They indicate the relative importance of the different items. Desirable properties of the item worst TTO scores are that the weights should not be too small—indicating an unimportant item—and, ideally, there should be no item which dominates other results.

TTO values for the VisQoL all-worst health state were assessed on a best health (DU = 0.00) to death (DU = 1.00) scale. The latter endpoint was used in preference to the combined VisQoL all-worst health state to minimize the cognitive burden upon interviewees. As the all-worst health state may be worse than death for respondents, the TTO protocol permitted this option.

## Discussion

The development of the VisQoL was motivated by the need to overcome two significant problems with existing QoL instruments.^9^ First, no vision-specific utility HRQoL instruments are currently available. Second, generic HRQoL utility instruments may not be sufficiently sensitive to vision-related QoL. Accurate measures of vision-related HRQoL are critical to assessing the value of eye-care and vision-related rehabilitation programs when resources for health care are limited.

The development of methods for the construction of the VisQoL MAU instrument was similarly motivated by a number of problems with existing MAU instruments. Historically, the construction of descriptive systems in MAU instruments (questions about health states) has been ad hoc. The calibration of utility scales has often been carried out with overly simple methodologies. Methods used to measure utilities and the models used to combine item scores into the multi-attribute health state utility differ significantly between instruments. Descriptive systems typically lack sensitivity in the good to best health range of the scale, limiting usefulness in some contexts (e.g. health promotion). Finally, utility instruments may be prone to the “focusing effect” where utility estimates may be biased downwards due to respondents’ focusing solely on the disutility of poor elements of health within a given health state, and failing to consider the utility of other elements.

The measurement of utility, by TTO or SG, may be carried out in one of two ways. In the “holistic” or composite approach to measurement, the relevant health states are described in a series of vignettes, or scenarios. These are then rated using the selected scaling instrument to obtain a utility index which is used to calculate QALYs. The construction of the health scenarios and the rating exercise both require surveys. Normally, patients who have experienced the health states are consulted for scenario construction, and a random sample of the population is used for the weighting survey. The second, “decomposed,” approach requires the preliminary construction of a generic multi-attribute utility (MAU) QoL instrument which is capable of describing numerous health states and which can then be weighted to assigning a utility score to each of these.

Both approaches have strengths and weaknesses. Holistic measurement permits a description which is tailored to a particular health state. Unique aspects of the health state, its context, its consequences, the process of health care delivery, risk and prognosis may all be included in the vignette. Validation of health state specific vignettes, however, is seldom, if ever, carried out. In contrast, the descriptive system of the MAU approach may be unable to capture many of the nuances of the health state and be incapable of capturing the importance of the process or context. However, this approach should, in principle, be based upon a descriptive system, the reliability and validity of which can be investigated using standard procedures. After construction, the use of an MAU instrument is cheap and easy and allows the rapid estimation of utilities in the context of a longitudinal trial.

Along with three other utility instruments, the VisQoL employs a multiplicative model for the combination of items into the final index of quality of life. For logical reasons this approach should be preferred to additive models, which are constrained so that the effect of any one dimension upon the quality of life must be relatively small. For example, under an additive model, suicidal depression cannot reduce a person to near suicide through the depression item alone.

However, the simple multiplicative model may overestimate the disutility of health states for two reasons. Firstly, the complexity of the concept of health-related quality of life makes it difficult to disaggregate it into its constituent parts without encountering redundancy—one element of a poor health state may be reflected in several items (i.e. there is double counting of this element). However, elimination of one set of such elements to eliminate double counting (for example deafness) may result in the omission of another, separate, element of health. To partially counter this potential problem, the disutility of the VisQoL all-worst health state was independently measured and the combined influence of items cannot exceed the value of the all-worst health state. Redundancy in the item scores will therefore not result in a score which exceeds the VisQoL all-worst score.

Secondly, the disutility of item worst health states are used as item importance weights. However, measurement of these weights is problematical. Those interviewed are asked to envisage and evaluate a state where one item is very bad but all other items are very good. As described above, steps were taken to reduce the likelihood of a “focusing effect” — the judgment of the entire health state by the single dimension.

## Conclusion

The 6-item VisQoL has excellent psychometric properties and is specifically designed to be sensitive to vision-related quality of life. It is the first instrument to permit the rapid estimation of utility values for use in economic evaluation of vision-related programs.

## Appendix: Visqol Items

1. Does my vision make it likely I will injure myself (i.e. when moving around the house, yard, neighborhood, or workplace)?
2. Does my vision make it difficult to cope with the demands in my life?
3. Does my vision affect my ability to have friendships?
4. Do I have difficulty organizing any assistance I may need?
5. Does my vision make it difficult to fulfill the roles I would like to fulfill in life (e.g. family roles, work roles, community roles etc)?
6. Does my vision affect my confidence to join in everyday activities?
